# Evaluation of differential effects of metformin treatment in obese children according to pubertal stage and genetic variations: study protocol for a randomized controlled trial

**DOI:** 10.1186/s13063-016-1403-4

**Published:** 2016-07-18

**Authors:** Belén Pastor-Villaescusa, Javier Caballero-Villarraso, M. Dolores Cañete, Raúl Hoyos, José Maldonado, Gloria Bueno, Rosaura Leis, Ángel Gil, Ramón Cañete, Concepción M. Aguilera

**Affiliations:** Department of Biochemistry and Molecular Biology II, Institute of Nutrition and Food Technology, Center of Biomedical Research Laboratory 123, University of Granada, Avenida del Conocimiento s/n. 18006 Armilla, Granada, Spain; Clinical Analysis Services. IMIBIC/Reina Sofía Hospital, Córdoba University, Córdoba, Spain; PAIDI CTS-329. Maimonides Institute of Biomedical Research of Córdoba (IMIBIC), Córdoba, Spain; Pediatric Department, Virgen de las Nieves University Hospital, Andalusian Health Service, Granada, Spain; Pediatric Gastroenterology and Nutrition Unit, Virgen de las Nieves University Hospital, Pediatric Department, University of Granada, Granada, Spain; Pediatric Department, Lozano Blesa University Clinical Hospital, University of Zaragoza, Zaragoza, Spain; Unit of Investigation in Nutrition, Growth and Human Development of Galicia, Pediatric Department, Clinic University Hospital of Santiago, University of Santiago de Compostela, Santiago de Compostela, Spain; Unit of Pediatric Endocrinology, Reina Sofia University Hospital, Córdoba, Spain; CIBER Fisiopatología de la Obesidad y la Nutrición (CIBEROBN), Madrid, Spain; Instituto de Investigación Biosanitaria ibs, Granada, Spain

**Keywords:** Metformin, Children, Obesity, Puberty, Lifestyle intervention, Microbiota, Polymorphisms

## Abstract

**Background:**

Overweight and obesity are considered to be serious public health problems. In pediatric populations, insulin resistance, dyslipidemia, and hypertension associated with obesity occur with increased frequencies. Metformin is an oral anti-hyperglycemic agent that has been demonstrated to be efficacious in the treatment of diabetic and non-diabetic obese adults. A considerable amount of pharmacogenetic research has demonstrated that genetic variation is one of the major factors affecting metformin response. Additionally, potential microbiota-mediated mechanisms of metformin effect have been recently described. However, scant work has been conducted in children, with no attention being paid to the potential effects of pubertal development. Thus, the main objective of the present study is to evaluate the effect of metformin treatment together with lifestyle recommendations in a randomized control trial (RCT) of obese children according to pubertal stage, genetic variants and signature of gut microbiota.

**Methods/design:**

This is a randomized, prospective, double-blind, placebo-controlled, multicenter trial, which is stratified by puberty and sex. Eighty pre-pubertal (40 boys and 40 girls) and 80 pubertal non-diabetic obese children (40 boys and 40 girls) are being recruited in four Spanish Clinical Hospitals. The inclusion criteria to participate in the RCT include a Body Mass Index (BMI) above the 95th percentile and age 7–14 years. The pubertal stage is determined based on the Tanner criteria. Participants are assigned to two groups in accordance with a randomization schedule and receive 1 g of metformin or placebo for six months in combination with healthy lifestyle recommendations in both groups. The primary outcomes include changes in the BMI Z score and the biomarkers associated with the early appearance of insulin resistance syndrome, inflammation, cardiovascular risk according of the presence of genetic determinants of metformin response, as well as possible modifications in microbiota.

**Discussion:**

This study will assess the differential response of metformin treatment at six months in pre-pubertal and pubertal obese children.

**Trial registration:**

Registered by European Clinical Trials Database (EudraCT, ID: 2010-023061-21) on 14 November 2011.

**Electronic supplementary material:**

The online version of this article (doi:10.1186/s13063-016-1403-4) contains supplementary material, which is available to authorized users.

## Background

The increased prevalence of obesity in pediatric populations is a public health problem [1]. Insulin resistance, impaired glucose tolerance, dyslipidemia, and hypertension are increased in children [2–4]. These metabolic alterations, which primarily result from obesity, begin in childhood and may manifest during adolescence or young adulthood, with diet and a sedentary lifestyle playing decisive roles [5]. In addition to a lifestyle intervention program, pharmacological treatments have been explored. Several drugs have been approved by the Food and Drug Administration (FDA) for the treatment of adult obesity. Currently, orlistat and sibutramine remain widely used in clinical practice in adults. Only orlistat has been approved for use in adolescents [6].

Metformin is an oral anti-hyperglycemic agent approved by the FDA to treat type 2 diabetes (T2D) in adults and children older than 10 years of age. Significant weight loss induced by metformin treatment has been demonstrated in both diabetic and non-diabetic obese adult patients [7]. In contrast, there is insufficient evidence regarding the effects of metformin in pediatric obesity. Several clinical trials have identified modest improvements following metformin treatment in insulin sensitivity in obese children with normal glucose tolerance [8–10], as well as a decrease in the BMI of obese adolescents [11]. In addition, metformin appears to improve lipid profiles in obese children [12, 13]. However, little is known regarding the effects of metformin, along with diet and exercise, on other measures associated with cardiovascular risk and inflammatory biomarkers. Six studies have evaluated the effects of doses of between 1000 and 2000 mg/day for 3–36 months in obese children and/or adolescents on inflammatory biomarkers related to obesity [10, 14–18]. These studies identified promising results, but did not follow a homogeneous distribution according to pubertal stage. Puberty is a very relevant confounding factor with a potential influence on insulin resistance development. Thus, randomized control trials (RCTs) with adequate statistical power appear necessary to enable the examination of these potential confounders [19], apart from a methodology based on a completely homogeneous distribution of factors, such as puberty and sex.

Furthermore, the composition of gut microbiota during early life has been proposed to influence the development of obesity and metabolic disease in children [20]. The scientific community emphasizes the need to disentangle gut microbiota signatures of specific human diseases from medication treatment success. Interestingly, the microbial mediation of the therapeutic effects of metformin through short-chain fatty acid production, as well as the potential microbiota-mediated mechanisms behind known intestinal adverse effects in the form of a relative increase in the abundance of *Escherichia* species have been demonstrated [21].

On the other hand, studies have shown variability in the therapeutic response of metformin treatment in T2D or obese patients. Variations in metformin response may reflect phenotypic differences in drug action or drug distribution. Genetic polymorphisms in drug uptake transporter genes have been increasingly recognized as a possible mechanism accounting for variation in drug response [22–24]. Therefore, the inclusion of genetic analyses in RCTs could be determinant to elucidate the variations in metformin response.

## Methods

### Objectives

In accordance with the previously discussed background, the present study has four main objectives: the first objective is to determine the efficacy of metformin in combination with a lifestyle intervention in reducing BMI in obese children compared with placebo after 6 months; the second objective is to evaluate the effects on insulin resistance inherent to metabolic syndrome; the third objective is to identify its effects on inflammatory, cardiovascular risk and oxidative stress biomarkers; the fourth objective is to evaluate the change of gut microbiota composition after treatment. Additionally, metformin differential response will be analyzed according to genetic polymorphisms in drug uptake transporter genes.

### Study design

**Fig. 1:**
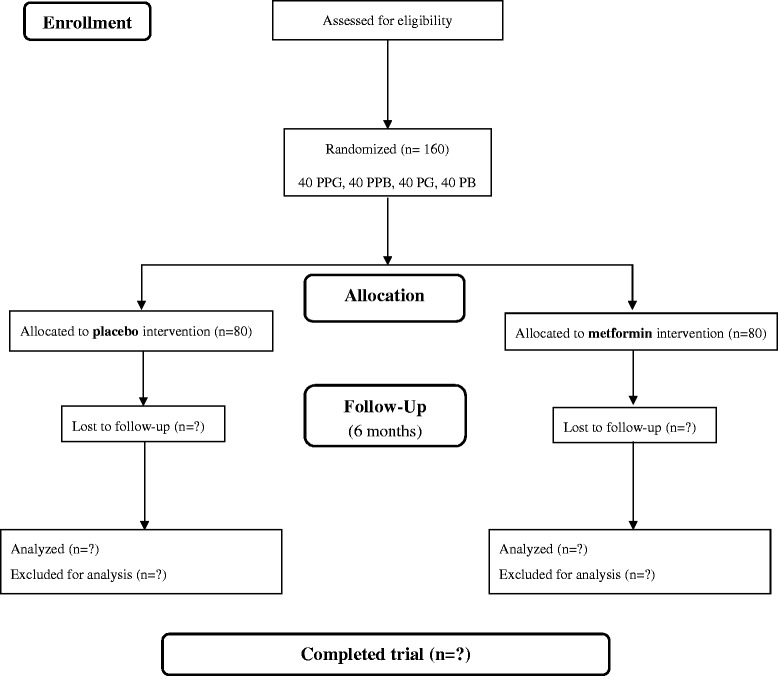
Participant flow diagram. *PPG* pre-pubertal girls; *PPB* pre-pubertal boys; *PG* pubertal girls; *PB* pubertal boys

The study is a multicenter investigation, stratified by sex and puberty (40 pre-pubertal girls, 40 pre-pubertal boys, 40 pubertal girls, and 40 pubertal boys). The patient distribution among groups is indicated in Fig. 1. The pubertal stage is determined based on the Tanner criteria [25]. This randomized, prospective, double-blind, placebo-controlled, multicenter trial is being conducted at four Spanish Hospitals: Córdoba, Granada, Santiago de Compostela and Zaragoza (Table 1).

**Table 1:** Distribution of patients according to center

Hospital	Total (160)
Reina Sofía (Córdoba)	20 pre-pubertal girls
	20 pre-pubertal boys
Virgen de las Nieves (Granada)	10 pre-pubertal girls
	10 pre-pubertal boys
	10 pubertal girls
	10 pubertal boys
Clínico Universitario (Santiago de Compostela)	10 pre-pubertal girls
	10 pre-pubertal boys
	10 pubertal girls
	10 pubertal boys
Lozano Blesa (Zaragoza)	20 pubertal girls
	20 pubertal boys

Children are randomly assigned to receive metformin or placebo for six months. Both treatments are administered during meals (to minimize gastrointestinal side effects and the risk of hypoglycemia). The participants’ parents are given a coded vial of pills that contains either metformin or placebo pills for two months. The concealed allocation process ensures that the participants and all investigators are unaware of the allocated treatment. All participants are offered lifestyle intervention advice at all visits.

The clinical hospitals that participate in the RCT form part of the Maternal and Child Health and Development (SAMID network). Moreover, the RCT has been registered in the European Clinical Trials Database on 14 November 2011 (EudraCT, ID: 2010-023061-21).

In accordance with the “International Conference on Harmonization of Technical Requirements for Registration of Pharmaceuticals for Human Use” Guide (ICH): “CPMP/ICH/291/96 Note for Guidance on General Considerations for Clinical Trials,” this is a phase III clinical trial because it involves a commercialized drug for which a new indication that is not included in its technical specifications will be investigated.

The CONSORT statement (Consolidated Standards of Reporting Trials) has been taken into account in the study design report, as well as for the abstract and the flow diagram (Fig. 1), thereby increasing the reporting quality for the RCT. Moreover, the Standard Protocol Items: Recommendations for Interventional Trials (SPIRIT) guidelines for this study protocol are attached in Additional file 1.

### Participants

The study subjects comprise patients referred from the Pediatric Endocrinology Unit of the corresponding study centers. Children are eligible for this RCT if they meet the inclusion criteria (Table 2). The data are collected in the pediatric outpatient clinics by dieticians. The data and samples are codified according to each center and subsequently centralized at the Institute of Nutrition and Food Technology “José Mataix” (INYTA) in Granada, Spain.

**Table 2:** Inclusion and exclusion criteria

Inclusion criteria	Exclusion criteria
BMI greater than the 95th percentile based on the standards set by Cole et al*.* [26]	Does not meet the established age
Age 7–14 years	Any previous underlying disease
No underlying disease or a history of pathology	Use of medication with metabolic side effects, such as diuretics, β-blockers, β-adrenergics, or corticoids
No medical treatment regarding weight control in the previous 12 months	Cases of monogenic obesity
No participation in a previous trial	Children subjected to long periods of rest
	Did not sign the informed consent

*BMI* Body Mass Index

### Randomization

The participants are assigned to metformin or placebo in accordance with a randomization schedule generated by the Pharmacy Service of the Virgen de las Nieves University Hospital in Granada, with MAS 100 version 2.1 software (Glaxo-Welcome, Madrid, Spain) by the Support Consortium to Biomedical Research Network (CAIBER). At each center, 50 % of the children are assigned to each group.

The drug presentation format for the metformin and placebo groups has the same appearance. The local physician responsible for the procedure is only aware of the box/bottle codes of the tablets (as well as the registration number of the participating child). These codes have a corresponding equivalent available only to the coordinating investigator who has the other data of pharmaceutical interest, such as the origin, lot number and manufacturing and packaging dates (as well as whether the drug is metformin or placebo). The aforementioned local physician is not aware of these data and thus assigns the participant to one study group, without knowledge of the primary treatment administered.

### Breaking of the study blind

All research staff are blinded for both the treatment allocation during the time of the study and the data analysis. The study blind will be broken after all analyses are completed. In the case of emergencies (e.g., serious adverse events, potentially unexpected serious adverse reactions), the blind will be broken following consultation with the principal investigator. These events will subsequently be reported to the Medical Ethics Committee.

### Interventions

**Fig. 2:**
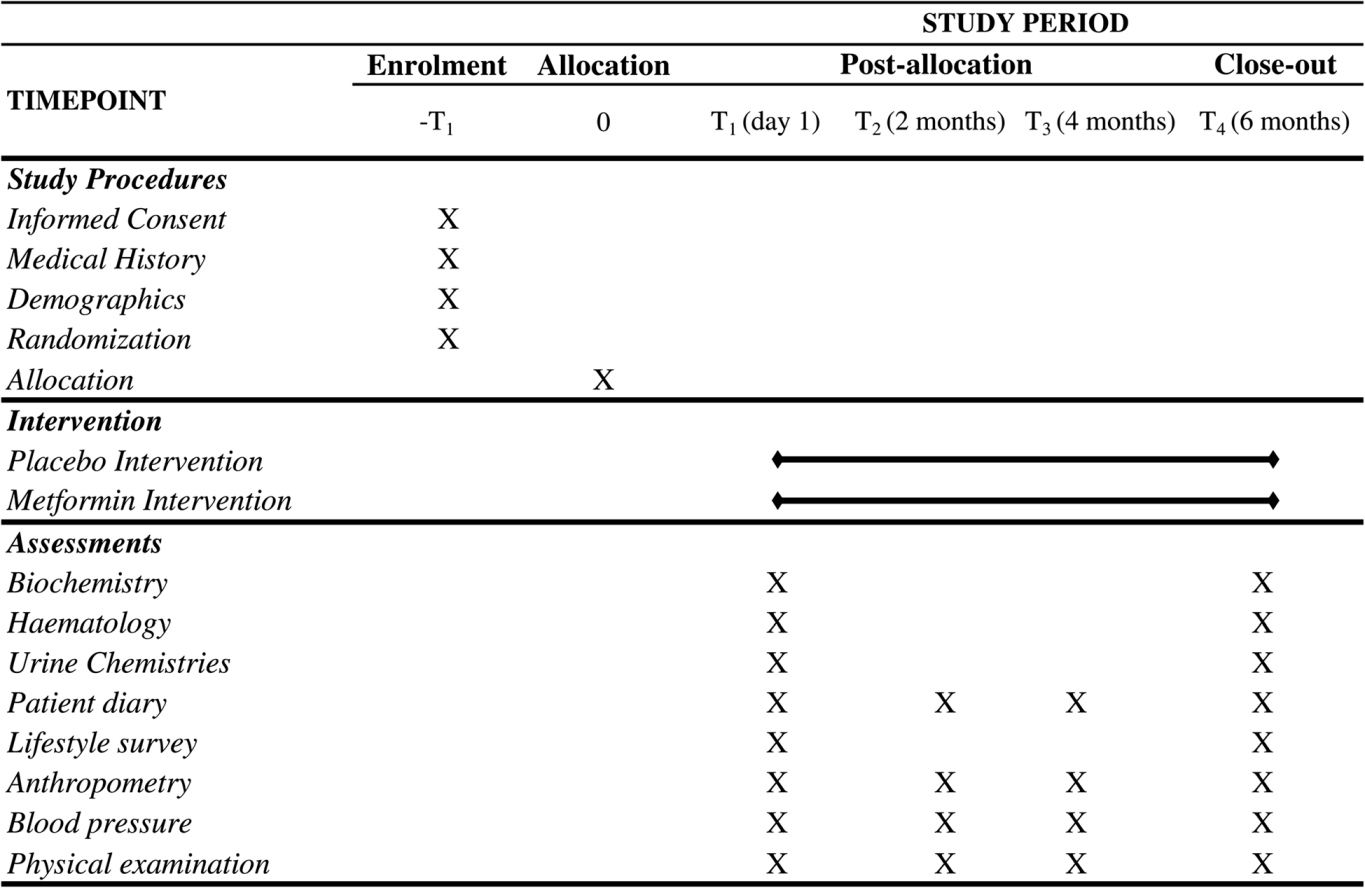
Activity schedule that may occur during each contact with the participant

The patients are instructed to taking a initial dose of 50 mg twice daily for 10 days, followed by 500 mg twice daily until the end of the treatment. The presentation comprises tablets in opaque, white plastic containers and a side label with 28 units. The dietician centers administer a food frequency questionnaire (FFQ) and a physical activity survey to all participants at the beginning and at the end of the trial. All participants are provided with standardized healthy lifestyle advice at the start of a one-on-one session, including a healthy diet and exercise advice sheet. The participants attend an initial trial baseline visit, followed by three additional visits at 2-month intervals (Fig. 2), which include anthropometric parameter and blood pressure (BP) assessments, as well as a physical examination. A medical history is obtained for each participant, including documentation of the family history.

To ensure the traceability of the treatments, a systematic record of the name of the pharmaceutical preparation, and the quantity and lot number dispensed to each subject are maintained in the corresponding data collection book. The data are updated according to the standard working procedures (SWPs) for the preparation and control of 500-mg metformin tablets and according to the SWPs for the control of pills, which are provided by the Hospital Pharmacy Department.

At the first visit, an extensive history is obtained. The duration of pregnancy, birth weight, neonatal feeding, use of medication, tobacco and alcohol, and the presence of maternal gestational diabetes are reported. Regarding family history, data on hypertension, obesity, hypercholesterolemia, cardiovascular disease, and *diabetes mellitus* in first-degree (parents) and second-degree (grandparents) family members are collected. Girls are asked whether and when they experienced menarche. The parental education level is recorded, as well as height and weight.

### Adverse effects and co-medication

To assess the safety of metformin administration, the primary evaluation criteria are the absence of the adverse effects (AEs) described. The patients are assessed regarding all symptoms at each visit to identify potential AEs and the use of co-medication in the previous two months. A contact number is provided to enable enquiries regarding any symptom perceived as adverse. At this number, the patient is informed about what to do in the event of an adverse reaction. They are also requested to suspend the medication. The following information is recorded: description, date of onset and end date, severity, assessment of relation to the study medication, other suspect drug or device and action taken. Follow-up information should be provided as necessary. The prominent AEs are as follow: diarrhea, nausea, blood in the stools, headache, dizziness, general discomfort, sleepiness, cold or flu, pharyngitis, otitis, allergic episode, lactic acidosis, urea increment, hypercreatininemia, hypertransaminasemia and vitamin B_12_ deficiency, as well as any other symptoms reported by the participants. The relationships of the AEs to the study medication will be assessed by a qualified medical investigator.

### Physical examination

The Clinical Units conduct a complete medical examination, which is performed with pubertal assessment. Measurements of arterial BP, calibrated by hand and in duplicate, and heart rate are obtained with the subjects in a seated position using a cuff appropriate for the arm circumference. The average BP values are expressed in mmHg, and the percentiles are determined and adjusted for sex and age according to the chart published by the National Heart, Lung and Blood Institute.

At every visit, an extended physical examination is performed by the research physician. This examination includes auscultation of the heart, lungs and abdomen and also abdominal palpation. Clinical signs may be identified, including the presence of acanthosis nigricans, hypertrichosis, striae, acne, adipomastia or hypogonadism.

### Monitoring of lifestyle

The dietician centers administer a FFQ and a physical activity survey to all participants at the beginning and at the end of the trial. Both questionnaires have been normalized by the IDEFICS and HELENA European Projects [26] and validated by the CTS-02203 Excellence Project of the Regional Government of Andalucía. The HELENA Study developed and tested a questionnaire for use among adolescents, based on the long format of the International Physical Activity Questionnaire (IPAQ) [27], which provided internationally comparable data [28].

### Blood, urine and fecal sampling

General biochemical analyses are performed at the participating hospitals following internationally accepted protocols.

Blood samples are obtained for biochemical and hematological screening tests between 08.30 and 10.30. Three milliliters of blood are collected at the beginning and at the end of the trial. The blood is drawn via the antecubital vein. Peripheral white blood cells (buffy coat) are taken for deoxyribonucleic acid (DNA) extraction. Moreover, a 1-ml urine sample is obtained for oxidation marker analysis. Children should not eat for 12 hours before the sampling. All samples are collected and stored frozen by the research staff. The samples will be analyzed in the clinical laboratory of each hospital, as well as the INYTA.

In order to studying the childrens’ gut microbiota, 100–200 g of fecal sample is collected in a sterile container by parents at the beginning and at the end of the trial. Immediately, all samples are stored frozen until to be analyzed at the INYTA.

### Adherence and tolerance

Adherence will be measured as a percentage using the following formula:

$$ \mathrm{Quick}=\left(\left(\mathrm{Pillsingested}{\textstyle \hbox{-}}\mathrm{pillsreturned}\right)/\mathrm{Pillspredicted}\right)\times 100 $$

These data will also be taken into account for statistical analysis as fixed effects. Tolerance is reported as the descriptive statistics of the adverse effects in relation to the achieved dosage level.

### Sample size

As previously indicated, the principal variable is the BMI *Z* score, on which the sample size calculation was based. Its standard deviation is 2.29 in the least favorable case (according to the tables by Cole et al. [29]), and a desired minimum difference of 2 points is expected. With an *α* error of 0.05, a β error of 0.20 and an estimated follow-up loss of 20 %, four groups in total are planned for the study: two groups of obese children (pre-pubertal and pubertal) treated with metformin and two groups of obese children (pre-pubertal and pubertal) treated with a placebo; there is a requirement of at least 40 patients per group (× four groups = 160 children total).

The clinical argument for the choice of the principal variable adheres to the fact that the obesity concept is based on the BMI, as the bibliography endorses. However, it should be noted that the BMI in childhood changes substantially with age [30]. Thus, age- and sex-specific cut-off points are needed to define pathology in children via means of a *Z* score [29] to obtain a more accurate value.

### Statistical analysis

Data will be analyzed using SPSS software version 22 for Windows. Descriptive statistics for all outcomes will be determined. A Kolmogorov-Smirnov test will be used to test data normality. Data that are not normally distributed will be transformed by means of a log10 or square root for analysis. The homogeneity of variances will be determined with a Levene’s test. Normally distributed data will be reported as mean ± standard deviation (SD) and nonparametric data as median (range). The analysis selected to determine the effect of treatment is a linear mixed effects model (LMM), for which the center is considered covariance to adjust the statistical analysis. The fixed effects include time, treatment, adherence, puberty, sex, the interactions *time × treatment*, *time × treatment × puberty* and *time × treatment × sex*. A Bonferroni test will be used to assess the specific differences between the treatments.

Regarding genetic variations, linear regression will be performed to analyze differences in principal variable changes between genotypes and to evaluate independent associated factors.

Moreover, the raw microbiologic data are reported as relative abundances. Differences among times, treatments and puberty are compared using the Mann-Whitney *U* test. Finally, the clustering of the colonic microbiota in both of groups is calculated by a principal component analysis.

### Interim analyses and stopping rules

Interim or preliminary analyses during the course of this RCT are not planned. In the event of subject withdrawal, a replacement or substitution is not planned; thus, these participants will be considered lost. The data associated with these subjects will subsequently be excluded from the statistical analysis. For this reason, the calculation of the sample size has included a potential loss of up to 20 %.

### Outcome measures

#### Anthropometry

Body weight (kg), height (cm) and waist circumference (cm) are measured via standardized procedures. The BMI and BMI *Z* score are calculated based on Spanish reference standards published by Sobradillo et al. [31]. Obesity is defined according to the BMI, with the age and sex-specific cut-off points proposed by Cole et al*.* [29] (BMI >95th percentile). Anthropometric measurements are obtained by a single examiner with the children barefoot and in their underwear. To obtain data on body composition, fat mass, lean mass and total body water are measured via bioimpedance technology using Tanita B18.

#### Biochemical analysis

The serum concentrations of glucose, lipids (total cholesterol, triglycerides (TG), high-density lipoprotein cholesterol (HDLc), and low-density lipoprotein cholesterol (LDLc)), apolipoprotein A1 (Apo-A1) and apolipoprotein B (Apo-B) are analyzed via spectrophotometry or a chemiluminescent microparticle immunoassay (CMIA) to measure the insulin concentration, according to auto-analyzers with standardized methods and both intra- and inter-laboratory control using internal and external quality control programmers at the Clinical Analysis Laboratory of each hospital. The Quantitative Insulin Sensitivity Check Index (QUICKI) and the homeostasis model assessment for insulin resistance (HOMA-IR) are calculated using the fasting plasma glucose and insulin values:

$$ \begin{array}{l}\mathrm{HOMA}=\mathrm{Fasting}\ \mathrm{insulin}\ \left(\upmu \mathrm{U}/\mathrm{ml}\right)\times \mathrm{fasting}\ \mathrm{glucose}\ \left(\mathrm{mmol}/\mathrm{l}\right)/22.5\\ {}1/\left(\mathrm{Log}\ \mathrm{fasting}\ \mathrm{insulin}\ \left(\upmu \mathrm{U}/\mathrm{ml}\right)+ \log\ \mathrm{fasting}\ \mathrm{glucose}\ \left(\mathrm{mg}/\mathrm{dl}\right)\right)\end{array} $$

#### Inflammation and cardiovascular risk biomarkers

Specific biomarkers of inflammation and cardiovascular risk, including adiponectin, leptin, resistin, myeloperoxidase (MPO), plasminogen activator inhibitor-1 (PAI-1), tumor necrosis factor-alpha (TNF-α), monocyte chemoattractant protein-1 (MCP-1), interleukin-6 (IL-6), interleukin-8 (IL-8), soluble intercellular adhesion molecule-1 (sICAM-1), soluble endothelial selectin (sE-Selectin) and soluble vascular adhesion molecule-1 (sVCAM-1) are analyzed in duplicate on a Luminex 200 system with the XMap technology (Luminex Corporation, Austin, TX, USA) and using human monoclonal antibodies (Milliplex Map Kit, Millipore, Billerica, MA, USA).

#### Oxidation biomarkers

The plasma total antioxidant capacity (TAC) is assessed with a spectrophotometric commercial antioxidant assay kit (Cayman, Ann Arbor, MI, USA), which is based on a colorimetric reaction. The oxidized LDL (Cayman, Ann Arbor, MI, USA), as well as the oxidative stress biomarkers isoprostane (Oxford Biomedical Research, Oxford, UK) and 8-hydroxy-2-deoxyguanosine (JaICA (Japan Institute for the Control of Ageing), Fukuroi, Shizuoka, Japan) are determined in duplicate via enzyme-linked immunosorbent assay (ELISA) in urine using a microplate reader BioTeK synergy HT.

#### Microbiota analysis

The fecal samples are subjected to extracting and purifying microbial DNA by a specific commercial DNA kit for purification (QIAamp DNA Stool Mini Kit, Quiagen, Barcelona, Spain). Quantification is conducted with a NanoDrop ND-1000 spectrophotometer (Thermo Fisher Scientific, Newark, DE, USA) in the Department of Microbiology, University Hospital San Cecilio (Granada, Spain). Polymerase chain reaction (PCR) is performed in a FastStart High Fidelity PCR System, dNTP Pack (Roche Applied Science). After PCR, amplicons are further purified using AMPure XP beads (Beckman-Coulter) to remove smaller fragments. DNA concentration and quality are measured using a Quant-iT™ PicoGreen® dsDNA Assay Kit. Afterwards, pyrosequencing of the PCR amplicons is performed using a Roche/454 GS Titanium technology platform (Roche, Branford, CT, USA). The MG-RAST (metagenomics analysis server) and the Ribosomal Database Project are used for the taxonomic analysis. Metagenomics data will be deposited in the publicly available repository MG-RAST (http://metagenomics.anl.gov/).

#### DNA isolation and genotyping

Genomic DNA is extracted from peripheral white blood cells (buffy coat) using the Zymo ZR-96 Quick-gDNA kit (Zymo Research Corporation, Irvine, CA, USA) according to the manufacturer’s instructions. Eleven single nucleotide polymorphisms (SNPs) previously described as being involved in the success of metformin treatment are being selected along several genes: the ataxia telangiectasia mutated (*ATM*, rs11212617); the glucokinase regulatory protein (*GCKR*, rs1260326); the serine-threonine kinase 11 (*STK11*, rs8111699); the peroxisome proliferator-activated receptor gamma, coactivator 1 alpha (*PPARGC1A*, rs2970852); the insulin-induced gene 2 (*INSIG2,* rs7566605); the neuronal growth regulator 1 (*NEGR1*, rs2815752); the solute carrier family 22 (organic cation transporter), member 1 (*SLC22A1*, rs622342); the solute carrier family 47 (multidrug and toxin extrusion), member 1 (*SLC47A1*, rs2289669); the transmembrane protein 18 (*TMEM18,* rs6548238), the potassium channel tetramerization domain containing 15 (*KCTD15*, rs29941); and the fat mass- and obesity-associated protein (*FTO*, rs9939609). Genotyping will be performed in duplicate using TaqMan® OpenArray® Genotyping Plates (ThermoFisher Scientific, Madrid, Spain).

## Discussion

The studies of the efficacy of metformin treatment in obese children and adolescents have been small, of short duration, or have used nonstandard doses of metformin and have produced inconclusive results. McDonagh et al. [19] examined the literature regarding obese children via a systematic review and meta-analysis and indicated the need for investigations of the effects of metformin treatment that consider the influence of potential confounding factors, such as puberty and sex. Similarly, trials with adequate statistical power that enable an examination of these potential confounders are mandatory. Nine trials mention the pubertal stage in their publications [8, 10, 11, 14, 16, 18, 32–34]. Although puberty has been considered in the study design by a limited number of authors, none of these authors used a homogenized sample division. Wilson et al*.* and Wiegand et al*.* predominantly recruited children who were further advanced in puberty [11, 33]. Yanovski et al*.* only considered pre-pubertal children or children in early puberty (Tanner I–III) [34], whereas Burgert et al. and Evia-Viscarra et al. only included adolescent volunteers [10, 14] and Freemark et al. included those at Tanner III [32]. Furthermore, the sample size has also been an important element: Srinivasan et al. recognized that the patient numbers were insufficient to statistically assess the effect of pubertal stage on the response to metformin therapy (*n* = 28, 14 pre-pubertal; 14 pubertal) [8]. Mauras et al. [16] evaluated metformin treatment with individual lifestyle coaching in pre- and pubertal children compared with a control group for six months. However, differences between the puberty groups based on metformin treatment were not identified. Furthermore, Kendall et al. [18] also examined the effects of metformin on obese children for six months. They did not identify a differential response to metformin according to the pubertal stage when they used a multifactorial regression analysis. This finding may also be explained by the small number of valid cases in the analysis segmented by puberty.

Additionally, during the last years the importance has been demonstrated of determining the specific contribution of the human gut microbiome to the pathogenesis of obesity to allow for the development of effective treatment strategies. Recently, an elegant study by Forslund et al. published in *Nature* identifies specific disease and drug signatures in the human gut microbiome of T2D patients treated with metformin [21]. By analyzing the dataset without stratifying for treatment regimens, they replicated the majority of previously reported results and showed a large divergence between the study populations. However, composition of gut microbiota from children treated with metformin has not been studied. Taking into account the proven role of the microbiota on childhood obesity development [20], the development of RCTs adjusting the effects according to the human gut microbiome is necessary.

Finally, a considerable amount of pharmacogenetic research has demonstrated that genetic variation is one of the major factors affecting metformin response [35]. Moreover, it has become increasingly clear that the pharmacokinetics of metformin are primarily determined by membrane transporters, including the plasma membrane monoamine transporter, the organic cation transporters (OCTs), the multidrug and toxin extrusion-1 transporter (MATE1), and the critical AMPK. In this RCT, we will analyze the genetic variants previously proved to determine the pharmacokinetics of metformin [36] and a differential response after treatment in obese subjects. Genes included are related to metformin transporters (*SLC22A1* and *SLC47A1*), AMPK and the gluconeogenesis pathway (*ATM*, *STK11* and *PPARGC1A*), insulin sensitivity *(GCKR* and *INSIG2*) and weight loss or weight regain predictors (*FTO*, *TMEM18, NEGR1* and *KTCD15*) Understanding how genetic variation affects metformin response will help to promote more effective use of the drug for the treatment of childhood obesity.

In view of this situation, our research could provide consolidated evidence regarding metformin’s effects on obese children, considering the pubertal stage via homogeneous Tanner stratification, as well as finding possible lines of action by metformin. Nevertheless, the study has several limitations, including the difficulty of assessing treatment compliance in children, as well as lifestyle changes. Moreover, the supervision of dietary habits and physical activity proves rather complicated. We are controlling for medication taken by means of the delivery and return of the bottles; however, we are aware that this strategy does not ensure accuracy regarding intervention compliance information.

### Trial status

Currently, the trial is ongoing and recruitment of participants continues.

## Abbreviations

AEs, adverse events; AMPK, AMP-activated protein kinase; Apo-A1, apolipoprotein A1; Apo-B, apolipoprotein B; *ATM*, ataxia telangiectasia mutated; BMI, body mass index; FFQ, food frequency questionnaire; CMIA, chemiluminescent microparticle immunoassay; ELISA, enzyme-linked immunosorbent assay; FDA, Food and Drug Administration; *GCKR*, glucokinase regulatory protein; HDLc, high-density lipoprotein cholesterol; HOMA-IR, homeostasis model assessment for insulin resistance; IL-6, interleukin-6; IL-8, interleukin-8; *INSIG2*, insulin-induced gene 2; IPAQ, physical activity questionnaire; *KCTD15*, potassium channel tetramerization domain containing 15; LDLc, low-density lipoprotein cholesterol; LMM, linear mixed effects model; *MATE1*, multidrug and toxin extrusion-1 transporter; MCP-1, monocyte chemoattractant protein-1; MG-RAST, metagenomics analysis server; MPO, myeloperoxidase; *NEGR1*, neuronal growth regulator 1; OCTs, organic cation transporters; PAI-1, plasminogen activator inhibitor-1; PPARGC1A, peroxisome proliferator-activated receptor gamma, coactivator 1 alpha; QUICKI, quantitative insulin sensitivity check index; RCT, randomized clinical trial; sE-selectin, soluble endothelial selectin; sICAM-1, soluble intercellular adhesion molecule-1; sVCAM-1, soluble vascular adhesion molecule-1; T2D, type 2 diabetes; TAC, total antioxidant capacity; TNF-α, tumor necrosis factor-alpha; *TMEM18*, transmembrane protein 18.

## Additional file

Additional file 1: SPIRIT 2013 Checklist (17.05.2016). File outlining how this study protocol meets the different guidelines from the SPIRIT 2013 Checklist. (PDF 48 kb)
